# Psychological treatments for excessive gaming: a systematic review and meta-analysis

**DOI:** 10.1038/s41598-022-24523-9

**Published:** 2022-11-28

**Authors:** Jueun Kim, Sunmin Lee, Dojin Lee, Sungryul Shim, Daniel Balva, Kee-Hong Choi, Jeanyung Chey, Suk-Ho Shin, Woo-Young Ahn

**Affiliations:** 1 Department of Psychology, Chungnam National University, W12-1, Daejeon, 34134 South Korea; 2 Department of Health and Medical Informatics, College of Health Sciences, Kyungnam University, Changwon, South Korea; 3 Department of Counseling Psychology, University of Georgia, Athens, GA USA; 4 School of Psychology, Korea University, Seoul, South Korea; 5 Department of Psychology, Seoul National University, Seoul, South Korea; 6 Dr. Shin’s Neuropsychiatric Clinic, Seoul, South Korea

**Keywords:** Psychology, Human behaviour

## Abstract

Despite widespread public interest in problematic gaming interventions, questions regarding the empirical status of treatment efficacy persist. We conducted pairwise and network meta-analyses based on 17 psychological intervention studies on excessive gaming (*n* = 745 participants). The pairwise meta-analysis showed that psychological interventions reduce excessive gaming more than the inactive control (standardized mean difference [SMD] = 1.70, 95% confidence interval [CI] 1.27 to 2.12) and active control (SMD = 0.88, 95% CI 0.21 to 1.56). The network meta-analysis showed that a combined treatment of Cognitive Behavioral Therapy (CBT) and Mindfulness was the most effective intervention in reducing excessive gaming, followed by a combined CBT and Family intervention, Mindfulness, and then CBT as a standalone treatment. Due to the limited number of included studies and resulting identified methodological concerns, the current results should be interpreted as preliminary to help support future research focused on excessive gaming interventions. Recommendations for improving the methodological rigor are also discussed.

## Introduction

Excessive gaming refers to an inability to control one’s gaming habits due to a significant immersion in games. Such an immersion may result in experienced difficulties in one’s daily life^1^, including health problems^2^, poor academic or job performance^3,4^, and poor social relationships^5^. Although there is debate regarding whether excessive gaming is a mental disorder, the 11th revision of the *International Classification of Diseases* (ICD-11) included Gaming Disorder as a disorder in 2019^6^. While there is no formal diagnosis for Gaming Disorder listed in the *Diagnostic and Statistical Manual of Mental Disorders, Fifth Edition* (DSM-5), the DSM-5 included Internet Gaming Disorder (IGD) as a condition for further study^7^. In the time since the DSM-5’s publication, research on excessive gaming has widely continued. Although gaming disorder’s prevalence appears to be considerably heterogeneous by country, results from a systematic review of 53 studies conducted between 2009 and 2019 indicated a global prevalence of excessive gaming of 3.05%^8^. More specifically, a recent study found that Egypt had the highest IGD prevalence rate of 10.9%, followed by Saudi Arabia (8.8%), Indonesia (6.1%), and India (3.8%) among medical students^9^.

While the demand for treatment of excessive gaming has increased in several countries^10^, standard treatment guidelines for problematic gaming are still lacking. For example, a survey in Australia and New Zealand revealed that psychiatrics— particularly child psychiatrists, reported greater frequency of excessive gaming in their practice, yet 43% of the 289 surveyed psychiatrists reported that they were not well informed of treatment modalities for managing excessive gaming^11^. Similarly, 87% of mental health professionals working in addiction-related institutions in Switzerland reported a significant need for professional training in excessive gaming interventions^12^. However, established services for the treatment of gaming remain scarce and disjointed.

Literature has identified a variety of treatments for excessive gaming, but no meta-analysis has yet been conducted on effectiveness of the indicated interventions. The only meta-analysis to date has focused on CBT^13^, and while results demonstrated excellent efficacy in reducing excessive gaming. However, the study did not compare the intervention with other treatment options. Given that gaming behavior is commonly affected by cognitive and behavioral factors as well as social and familial factors^14–16^, it would also be important to examine the effectiveness of treatment approaches that reflect social and familial influences. While two systematic reviews examined diverse therapeutic approaches, they primarily reported methodological concerns of the current literature and did not assess the weight of evidence^17,18^. Given that studies in this area are rapidly evolving and studies employing rigorous methodological approaches have since emerged^19,20^, a meta-analytic study that analyzes and synthesizes the current stage of methodological limitations while also providing a comprehensive comparison of intervention options is warranted.

In conducting such a study, undertaking a traditional pairwise meta-analysis is vital to assess overall effectiveness of diverse interventions. Particularly, moderator and subgroup analyses in pairwise meta-analysis provide necessary information as to whether effect sizes vary as a function of study characteristics. Furthermore, to obtain a better understanding of the superiority and inferiority of all clinical trials in excessive gaming psychological interventions, it is useful to employ a network meta-analysis, which allows for a ranking and hierarchy of the included interventions. While a traditional pair-wise analysis synthesizes direct evidence of one intervention compared with one control condition, a network meta-analysis incorporates multiple comparisons in one analysis regardless of whether the original studies used them as control groups. It enters all treatment and control arms of each study, and makes estimates of the differences in interventions by using direct evidence (e.g., direct estimates where two interventions were compared) and indirect evidence (e.g., generated comparisons between interventions from evidence loops in a network^21^. Recent meta-analytic studies on treatments for other health concerns and disorders have used this analysis to optimize all available evidence and build treatment hierarchies^22–24^.

In this study, the authors used a traditional pairwise meta-analysis and network meta-analysis to clarify the overall and relative effectiveness of psychological treatments for excessive gaming. The authors also conducted a moderator analysis to examine potential differences in treatment efficacy between Randomized Controlled Trials (RCTs) and non-RCTs, age groups, regions, and research qualities. Finally, the authors examined follow-up treatment efficacy and treatment effectiveness on common comorbid symptoms and characteristics (e.g., depression, anxiety, and impulsivity).

## Methods

The protocol for this review has been registered in the International Prospective Register of Systematic Review (PROSPERO 2021: CRD 42021231205) and is available for review via the following link: https://www.crd.york.ac.uk/PROSPERO/display_record.php?RecordID=231205. Preferred Reporting Items for Systematic Reviews and Meta-Analyses (PRISMA) network meta-analysis checklist^25^ is included in Supplementary Material 1.

### Identification and selection of studies

The authors searched seven databases, which included ProQuest, PubMed, Scopus, Web of Science, PsycINFO, Research Information Sharing Service (RISS), and DBpia. Given that a substantial number of studies have been published particularly in East Asia and exclusion of literature from the area in languages other than English has been discussed as a major limitation in previous reviews^17,18^, the authors gave special attention to gaming treatment studies in English and other languages from that geographical area. Additionally, the authors searched Google Scholar to ensure that no studies were accidentally excluded. The authors conducted extensive searches for studies published in peer-reviewed journals between the first available year (year 2002) and October 31, 2022, using the following search terms: “internet”, or “video”, or “online”, or “computer”, and “game”, or “games”, or “gaming”, and “addiction”, or “addictions”, or “disorder”, “disorders”, or “problem”, or “problems”, or “problematic”, or “disease”, or “diseases”, or “excessive”, or “pathological”, or “addicted”, and “treatment”, or “treatments”, or “intervention”, or “interventions”, or “efficacy”, or “effectiveness”, or “effective”, or “clinical”, or “therapy”, or “therapies”. Search strategies applied to each database is provided in Supplementary Material 2.

The authors included studies that recruited individuals who were excessively engaging in gaming, according to cutoff scores for different game addiction scales. Since there is not yet an existing consensus on operational definitions for excessive gaming, the authors included studies that recruited individuals who met high-risk cutoff score according to the scales used in each respective study (e.g., Internet Addiction Test [modified in game environments] > 70). The authors also sought studies that provided pretest and posttest scores from the game addiction scales in both the intervention and control groups. Studies meeting the following criteria were excluded: (a) the study targeted excessive Internet use but did not exactly target excessive gaming; (b) the study provided a prevention program rather than an intervention program; (c) the study provided insufficient data to perform an analysis of the effect sizes and follow-up contact to the authors of such studies did not yield the information necessary for inclusion within this paper; and (d) the study conducted undefinable types of intervention with unclear psychological orientations (e.g., art therapy with an undefined psychological intervention, fitness programs, etc.).

Two authors (D.L. and S.L.) independently screened the titles and abstracts of articles identified by the electronic searches and excluded irrelevant studies. A content expert (J.K.) examined the intervention descriptions to determine intervention types that were eligible for this review. All treatments were primarily classified based on the treatment theory, protocol, and descriptions about the procedures presented in each paper. D.L. and S.L.—both of whom have been in clinical training for 2 years categorized treatment type, to which J.K., a licensed psychologist, cross-checked and confirmed the categorization. The authors resolved disagreements through discussion. The specific example of intervention type classification is provided in Supplementary Material 3.

### Risk of bias and data extraction

Three independent authors assessed the following risks of bias among the included studies. The authors used the Risk of Bias 2.0 (RoB 2) tool for RCT studies and the Risk Of Bias In Non-Randomized Studies of Intervention (ROBINS-I) tool for non-RCT studies. The RoB 2 evaluates biases of (a) randomization processes; (b) deviations from intended interventions; (c) missing outcome data; (d) measurement of the outcome; and (e) selection of the reported result, and it categorizes the risk of bias in each dimension into three levels (low risk, moderate risk, and high risk). The ROBINS-I evaluates biases of (a) confounding variables; (b) selection of participants; (c) classification of interventions; (d) deviations from intended interventions; (e) missing data; (f) measurement of outcomes; and (g) selection of the reported result, and it categorizes the risk of bias in each dimension into five levels (low risk, moderate risk, serious risk, critical risk, and no information). After two authors (D.L. and S.L.) assessed each study, another author (J.K.) cross-checked the assessment.

For each study, the authors collected descriptive data, which included the sample size as well as participants’ ages, and regions where the studies were conducted. The authors also collected clinical data, including whether the study design was a RCT, types of treatment and control, treatment duration, and the number of treatment sessions. Finally, the authors collected data on the follow-up periods and the measurement tools used in each study.

### Data analysis

The authors employed separate pairwise meta-analyses in active control and inactive control studies using R-package “meta”^26^ and employed a random-effects model due to expected heterogeneity among studies. A random-effects model assumes that included studies comprise random samples from the larger population and attempt to generalize findings^27^. The authors categorized inactive control groups including *no treatment* and *wait-list control* and categorized active control groups including *pseudo training* (e.g., a classic stimulus-control compatibility training) and other types of psychological interventions (e.g., Behavioral Therapy, CBT, etc.). The authors also used the bias-corrected standardized mean change score (Hedges’ *g*) due to small sample sizes with the corresponding 95% confidence interval^28^. The authors’ primary effectiveness outcome was a mean score change on game addiction scales from pre-treatment to post-treatment. Hedges’ g effect sizes were interpreted as small (*g* = 0.15), medium (*g* = 0.40) and large (*g* = 0.75), as suggested by Cohen ^29^. The authors used a conservative estimate of *r* = 0.70 for the correlation between pre-and post-treatment measures^30^, and to test heterogeneity, the authors calculated Higgins’ I^2^, which is the percentage of variability in effect estimates due to heterogeneity among studies rather than chance. I^2^ > 75% is considered substantial heterogeneity^31^.

The authors conducted moderator analyses as a function of RCT status (RCT versus non-RCT), age group (adolescents versus adults), region (Eastern versus Western), and research quality (high versus low). The authors divided high versus low quality studies using median values of research quality scores (RCT: low [0–2] versus high [3–5], non-RCT: low [0–4] versus high [5]). The authors calculated Cochran’s Q for heterogeneity: A significant Q value indicates a potentially important moderator variable. For the subgroup analyses of follow-up periods and other outcomes, the authors conducted separate pairwise analyses in 1- to 3-month follow-up studies and in 4- to 6-month follow-up studies and separate analyses in depression, anxiety, and impulsivity outcome studies.

The authors sought to further explore relative effectiveness of treatment types and performed a frequentist network meta-analysis using the R-package “netmeta” 4.0.4 version ^26^. To examine whether transitivity and consistency assumptions for network meta-analysis were met, the authors assessed global and local inconsistency. To test network heterogeneity, the authors calculated Cochran’s Q to compare the effect of a single study with the pooled effect of the entire study. The authors drew the geometry plot of the network meta-analysis through the netgraph function in “netmeta”, and the thicker lines between the treatments indicated a greater number of studies.

The authors presented the treatment rankings based on estimates using the surface area under the cumulative ranking curve (SUCRA)^32^. The SUCRA ranged from 0 to 100%, with higher scores indicating greater probability of more optimal treatment. The authors also generated a league table to present relative effectiveness between all possible comparisons between treatments. When weighted mean difference for pairwise comparisons is bigger than 0, it favors the column-defining treatment. Finally, funnel plots and Egger’s test were used to examine publication bias.

## Results

### Included studies and their characteristics

**Figure 1:**
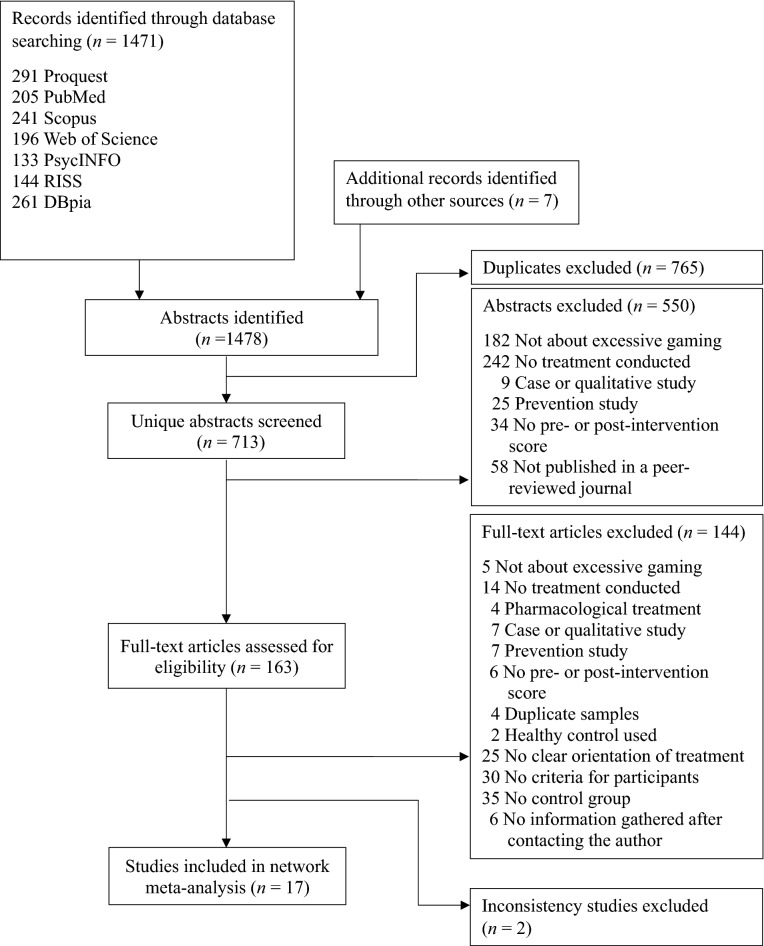
Flow diagram of the study selection process.

Figure 1 presents the flow diagram of the study selection process. The authors identified 1471 abstracts in electronic searches and identified an additional seven abstracts through secondary/manual searches (total *n* = 1478). After excluding duplicates (*n* = 765) and studies that did not meet the inclusion criteria based on the study abstract (*n* = 550), the authors retrieved studies with potential to meet the inclusion criteria for full review (*n* = 163). Of these, 144 studies were excluded due to not meeting inclusion criteria based on full-text articles, leaving 19 remaining studies. Of the 19, two studies did meet this paper’s inclusion criteria but were excluded from this network meta-analysis^33,34^ because the consistency assumption between direct and indirect estimates was not met at the time of this study's consideration based on previous studies^35,36^. Therefore, a total of 17 studies were included in this network meta-analysis, covering a total of 745 participants^36^.

Table 1 lists the characteristics of the 17 included studies. CBT (*n* = 4), Behavioral Treatment (BT) + Mindfulness (*n* = 4), and BT only (*n* = 4) were most frequently studied, followed by CBT + Family Intervention (*n* = 1), CBT + Mindfulness (*n* = 1), virtual reality BT (*n* = 1), Mindfulness (*n* = 1), and Motivational Interviewing (MI) + BT (*n* = 1). Seven studies were conducted in Korea and six were conducted in China, followed by Germany and Austria (*n* = 1), Spain (*n* = 1), the United States (*n* = 1), and the Philippines (*n* = 1). Twelve articles were written in English, and five articles were written in a language other than English. Nine studies conducted a follow-up assessment with periods ranging from one to three months, and two studies conducted a follow-up assessment with periods ranging four to six months. In one study^20^, the authors described their 6-month follow-up but did not present their outcome value, and thus only two studies were included in the four- to six-month follow-up analysis. Among the 17 included studies, eight had no treatment control group, five had an active control group (e.g., pseudo training, BT, and CBT), and four had a wait-list control group. Seven of the studies were RCT studies, and 10 were non-RCT studies.

**Table 1:** Characteristics of included studies.

Author and year	N total (Trt N/Ctrl N)	Average age (male %)	Region	RCT	Type of treatment	Treatment duration (# of session)	Type of control	Follow-up	Outcome measurement
Kuriala and Reyes(2020)^51^	40 (20/20)	16–19 (n.r)	Philippines	Yes	CBT + Mndfulness	5wks (10)	No treatment	None	IGDS9-SE
Li et al. (2017)^52^	30 (15/15)	25 (80%)	US	Yes	Mndfulness	8 wks. (8)	CBT	3 mos.	DSM-5
Torres-Rodriguez et al. (2018)^56^	31 (16/15)	15 (100%)	Spain	No	CBT + Family	24 wks. (22)	CBT	3 mos.	IGD-20
Wölfling et al. (2019)^20^	143 (72/71)	26 (100%)	Germany & Austria	Yes	CBT	15 wks. (15)	Wait-list control	6 mos.	AICA-C (BDI)
Kang and Son (2010)^57^	14 (7/7)	15 (60%)	Korea	No	CBT	5 wks. (10)	No treatment	None	IGADS
Lee and An (2002)^58^	72 (42/30)	14 (55%)	Korea	No	CBT	9 wks. (9)	Wait-list control	2 mos.	IGADS (CDI, BIS, STAI)
Lee and Son (2008)^59^	27 (13/14)	16–19 (n.r.)	Korea	No	CBT	12 wks. (12)	BT	2 mos.	IAT (BDI)
Deng et al. (2017)^60^	58^a^ (44/19)	21 (100%)	China	No	BT + mindfulness	6 wks. (6)	wait-list control	3, 6 mos.	POGUS (BDI, BIS)
Liu et al. (2020)^39^	36^a^ (20/16)	22 (100%)	China	No	BT + mindfulness	6 wks. (6)	No treatment	3, 6 mos.	CIAS
Zhang et al. (2016a)^61^	36^a^ (20/16)	21 (100%)	China	No	BT + Mindfulness	6 wks. (6)	No treatment	None	CIAS
Zhang et al. (2016b)^62^	40^a^ (23/17)	21 (100%)	China	No	BT + Mndfulness	6 wks. (6)	No treatment	None	CIAS
Park et al. (2016)^53^	24^a^ (12/12)	23 (100%)	Korea	Yes	Virtual reality BT	4 wks. (8)	CBT	None	YIAS
Ju et al. (2011)^63^	24 (12/12)	13 (79%)	Korea	No	MI + BT	4 wks. (8)	No treatment	None	K- index
Zheng et al. (2022)^54^	40 (20/20)	17 (100%)	China	Yes	BT	2 wks. (15)	No treatment	1 mo.	OGAS (BIS, DASS)
Choi and Son (2011)^55^	20 (10/10)	19–27(47%)	Korea	Yes	BT	10 wks. (10)	Wait-list control	1 mo.	IGADS (EII)
He et al. (2021)^19^	48 (24/24)	20 (19%)	China	Yes	BT	n.r. (4)	Pseudo training	None	DSM-5 (BDI, BAI, BIS)
Pyo and Lee (2004)^64^	32 (16/16)	12 (88%)	Korea	No	BT	n.r. (8)	No treatment	6 wks.	IGADS

*AICA-C* Assessment of Internet and Computer game Addiction Checklist, *BAI* Beck Anxiety Inventory, *BDI* Beck Depression Inventory, *BIS* Barratt Impulsiveness Scale, *BT* Behavioral Therapy, *CBT* Cognitive Behavioral Therapy, *CDI* Children’s Depression Inventory, *CIAS* Chinese Internet Addiction Scale, *Ctrl N* sample size in control group, *DASS-21* The Depression Anxiety stress scale-21), *DSM-5* Diagnostic and Statistical Manual of Mental Disorders, Fifth Edition (endorsed by participants), *EII* Eysenck’s Impulsivity Inventory, *Family* Family Intervention, *IAT* Internet Addiction Test, *IGADS* Internet Game Addiction Diagnostic Scale, *IGD-20* Internet Gaming Disorder test-20, *IGDS9-SF* Internet Gaming Disorder Scale-Short-Form, *KGAS* Korean Game Addiction Scale for Adults, *K-index* Korean Internet Addiction Scale, *mo.* month, *mos.* months, *MI* motivational interviewing, *n.r.* not reported, *OGAS* online game addiction scale, *POGUS* Problematic Online Game Use Scale, *RCT* randomized controlled trial, *STAI* The State-Trait Anxiety Inventory, *Trt N* sample size in intervention group, *UPPS-P* Urgency, Premeditation, Perseverance, Sensation seeking, Positive urgency, impulsive behavior scale, *wks.* weeks, *YIAS* Young’s Internet Addiction Scale.

### Pairwise meta-analysis

**Figure 2:**
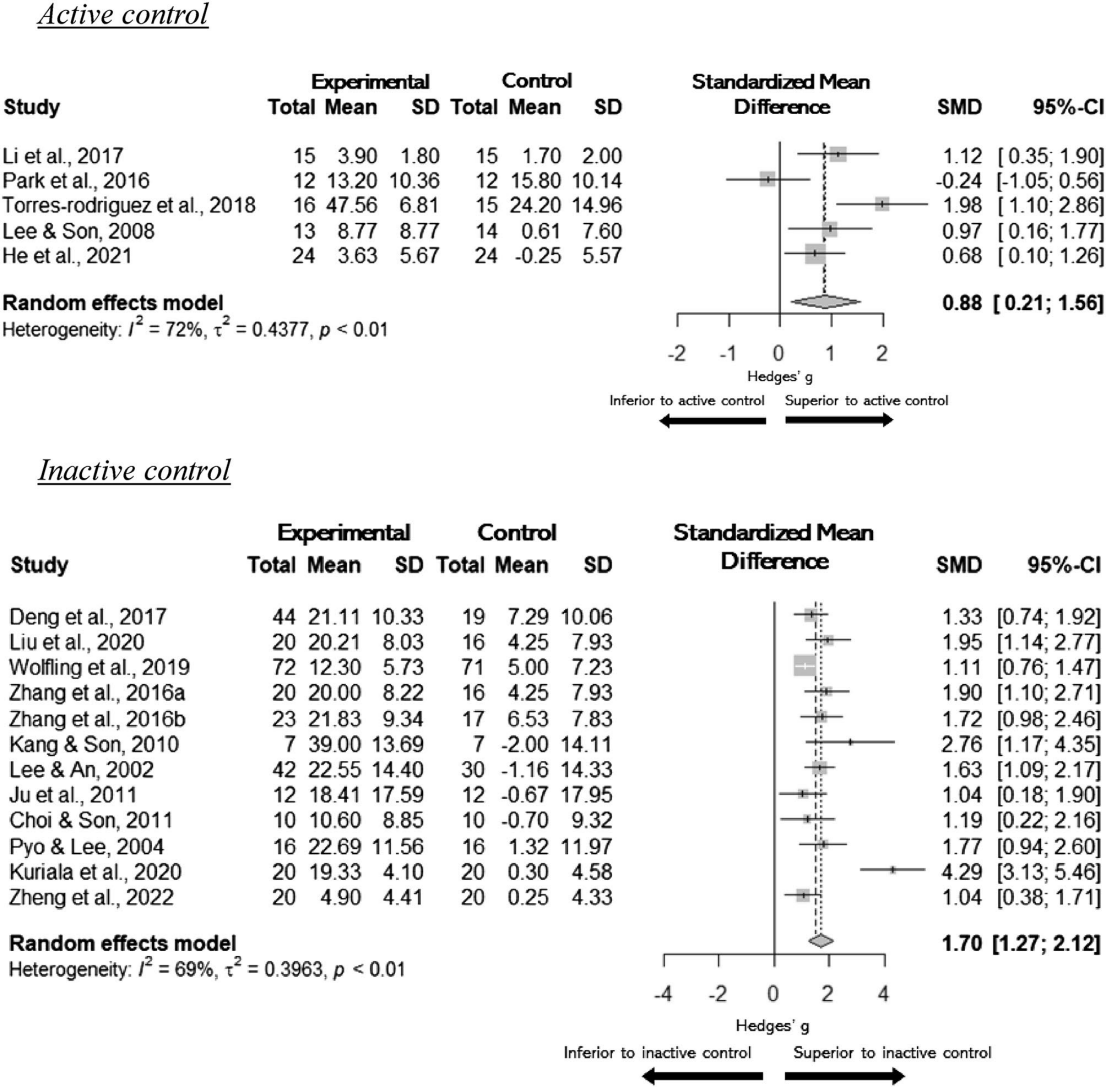
Pairwise Meta-analysis. Psychological treatment effects on excessive gaming by comparison group type (active and inactive controls). *SMD* standardized mean difference, *SD* standard deviation, *CI* confidence interval, I^2 ^= Higgins' I^2^.

The results of meta-analyses showed a large effect of all psychological treatments when compared to any type of comparison groups (*n* = 17, *g* = 1.47, 95% CI [1.07, 1.86]). The treatment effects were separately provided according to active versus inactive comparison groups in Fig. 2. The effects of psychological treatments were large when compared to the active control (*n* = 5, *g* = 0.88, 95% CI [0.21, 1.56]) or inactive control (*n* = 12, *g* = 1.70, 95% CI: [1.27, 2.12]). Substantial heterogeneity was evident in studies that were compared to both the active controls (I^2^ = 72%, < 0.01) and inactive controls at *p*-value level of 0.05 (I^2^ = 69%, *p* < 0.001).

#### Moderator analysis

As shown in Table 2, the moderator analysis suggested that effect sizes were larger in non-RCT studies (*n* = 10, *g* = 1.60, 95% CI [1.36, 1.84]) than RCT studies (*n* = 7, *g* = 1.26, 95% CI [0.30, 2.23]). However, the results of a Q-test for heterogeneity yielded insignificant results (Q = 0.44, df[Q] = 1, *p* = 0.51), indicating that no statistically significant difference in treatment efficacy at *p* level of 0.05 between RCT and non-RCT studies.

**Table 2:** Moderator and subgroup analysis.

**Moderator analysis**						
Variable	Group (range)	N	Hedges’ *g*	95% CI	Z-value	Q-test
RCT status	Non-RCT	10	1.60	[1.36, 1.84]	13.01***	0.44
	RCT	7	1.26	[0.30, 2.23]	2.58**	
Age group	Adults (19–52)	9	1.19	[0.77, 1.61]	5.55***	2.39
	Adolescents (11–18)	8	1.85	[1.12, 2.57]	5.01***	
Region	Eastern	14	1.36	[1.16, 1.57]	5.95***	0.40
	Western	3	1.29	[0.85, 1.73]	5.71***	
Quality (RCT)	High quality (3–5)	3	0.58	[− 0.17, 1.34]	1.52	2.25
	Low quality (0–2)	4	1.87	[0.37, 3.36]	2.44*	
Quality (Non-RCT)	High quality (5)	5	1.37	[1.02, 1.72]	7.59***	3.06
	Low quality (0–4)	5	1.80	[1.47, 2.13]	10.70***	

**Subgroup analysis**						
Variable	Subgroup	N	Hedges’ *g*	95% CI	Z-value	P-value
Follow-up period	1–3 months	9	1.54	[0.87, 2.21]	4.53***	< 0.001
	4–6 months	2	1.23	[0.77, 1.68]	5.31***	< 0.001
Other outcomes	Depression	6	0.52	[0.22, 0.81]	3.43***	< 0.001
	Impulsivity	5	0.26	[− 0.14, 0.67]	1.27	0.20
	Anxiety	3	0.60	[0.11, 1.08]	2.41*	0.02

*N* =  number of studies, *CI* = confidence interval, RCT randomized controlled trial.

*p < 0.05, **p < 0.01, ***p < 0.001.

The results of Q-test for heterogeneity did not yield any significant results, indicating no significant differences in treatment efficacy between adults and adolescents (Q = 2.39, df[Q] = 1, *p* = 0.12), Western and Eastern regions (Q = 0.40, df[Q] = 1, *p* = 0.53), or low and high research qualities among RCT studies (Q = 2.25, df[Q] = 1, *p* = 0.13) and non-RCT studies (Q = 3.06, df[Q] = 1, *p* = 0.08).

#### Subgroup analysis

The results demonstrated that the treatment effect was Hedges’ *g* = 1.54 (95% CI [0.87, 2.21]) at 1-to-3-month follow-up and Hedges’ *g* = 1.23 (95% CI [0.77, 1.68]) 4- to-6-month follow-up. The results also showed that the treatment for excessive gaming was also effective on depression and anxiety. Specifically, treatment on depression was Hedges’ *g* = 0.52 (95% CI: [0.22, 0.81], *p* < 0.001), and anxiety was Hedges’ *g* = 0.60 (95% CI [0.11, 1.08], *p* = 0.02), which are medium and significant effects. However, the effect on impulsivity was insignificant, Hedges’ *g* = 0.26 (95% CI [− 0.14, 0.67], *p* = 0.20).

### Network meta-analysis

**Figure 3:**
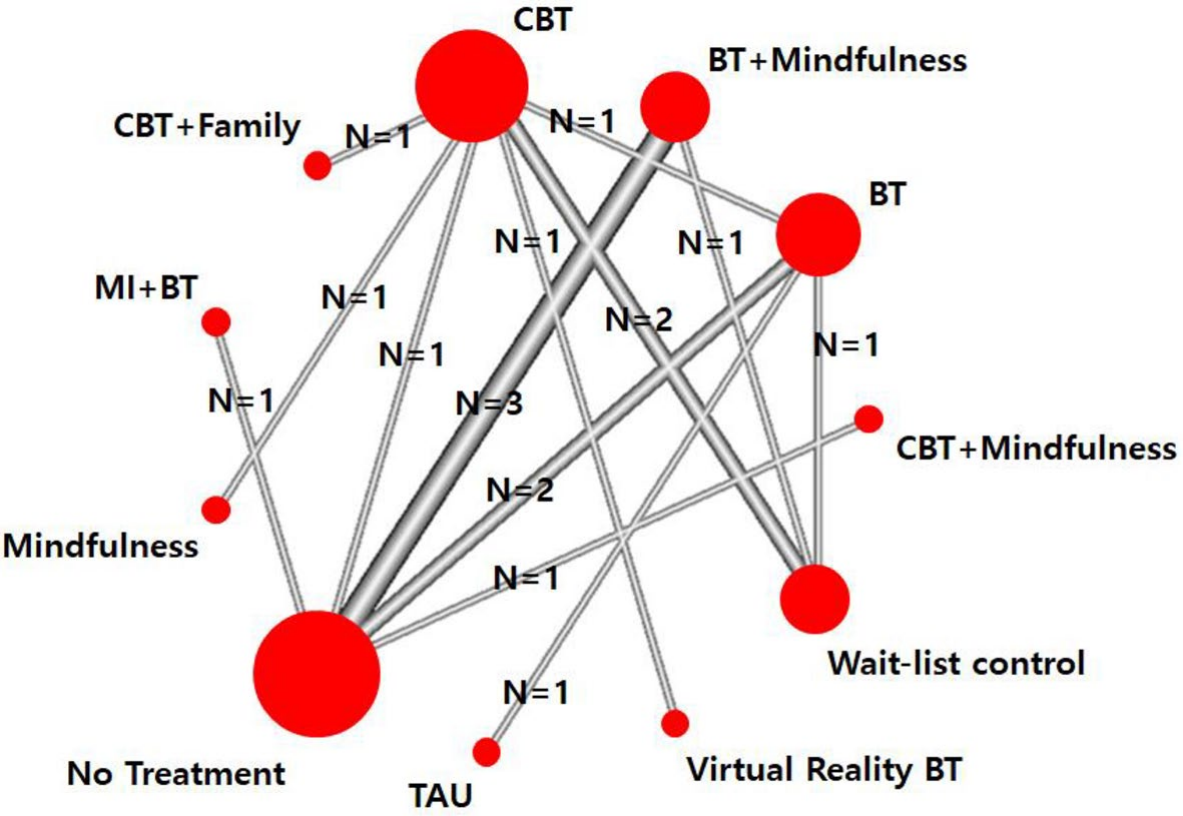
Network plot for excessive gaming interventions. Width of lines and size of circles are proportional to the number of studies in each comparison. *BT* behavioral therapy, *CBT* cognitive behavioral therapy, *Family* family intervention, *MI* motivational interviewing, *TAU* treatment as usual.

As shown in Fig. 3, a network plot represents a connected network of eight intervention types (CBT, BT + Mindfulness, BT, Virtual Reality BT, CBT + Mindfulness, CBT + Family, MI + BT, and Mindfulness) and three control group types (wait-list control, no treatment, treatment as usual). The widest width of nodes was observed when comparing BT + Mindfulness and no treatment, indicating that those two modules were most frequently compared. No evidence of global inconsistency based on a random effects design-by-treatment interaction model was found (Q = 8.5, df[Q] = 7, *p* = 0.29). Further, local tests of loop-specific inconsistency did not demonstrate inconsistency, indicating that the results from the direct and indirect estimates were largely in agreement (*p* = 0.12- 0.78).

**Figure 4:**
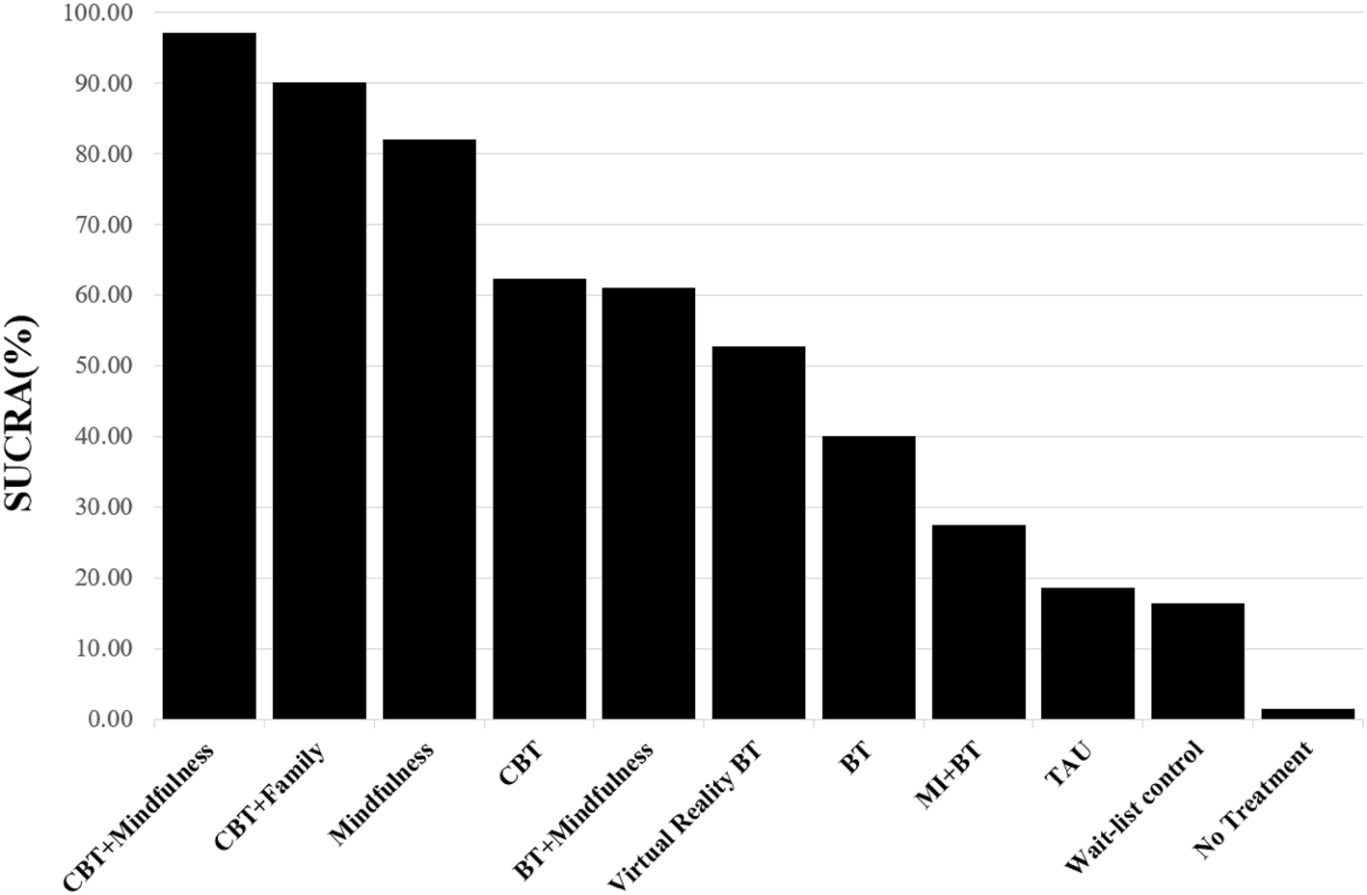
Surface under the cumulative ranking curve (SUCRA) rankogram of excessive gaming. *BT* behavioral therapy, *CBT* cognitive behavioral therapy, *Family* family intervention, *MI* motivational interviewing, *TAU* treatment as usual.

As shown in Fig. 4, according to SUCRA, a combined intervention of CBT and Mindfulness ranked as the most optimal treatment (SUCRA = 97.1%) and demonstrated the largest probability of effectiveness when compared to and averaged over all competing treatments. A combined treatment of CBT and Family intervention ranked second (SUCRA = 90.2%), and Mindfulness intervention ranked third (SUCRA = 82.1%). As shown in Table 3, according to league table, CBT + Mindfulness intervention showed positive weighted mean difference values in the lower diagonal, indicating greater effectiveness over all other interventions. The CBT + Mindfulness intervention was more effective than CBT + Family or Mindfulness interventions, but their differences were not significant (weighted mean differences = 0.23–1.11, 95% CI [− 1.39 to 2.68]). The top three ranked interventions (e.g., CBT + Mindfulness, CBT + Family intervention, and Mindfulness in a row) were statistically significantly superior to CBT as a standalone treatment as well as the rest of treatments.

**Table 3:**
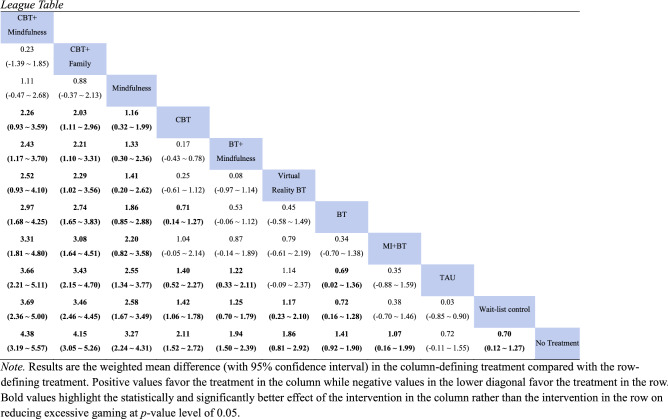
League table.

### Risk of bias

**Figure 5:**
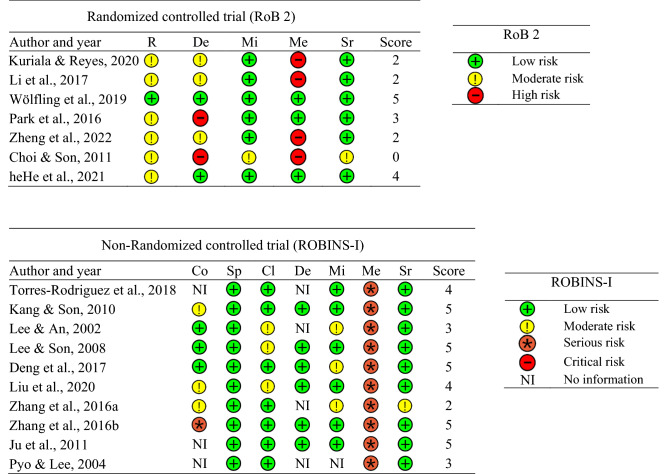
Overview of risk of bias results across all included studies. *Cl* bias in classification of interventions, *Co* bias due to confounding, *De* bias due to deviations from intended interventions, *Me* bias in measurement of the outcome, *Mi* bias due to missing outcome data, *R* bias arising from the randomization process, *RoB* risk of bias, *ROBINS-I* risk of bias in non-randomized studies of intervention, *Sp* bias in selection of participants in the study, *Sr* bias in selection of the reported result.

Figure 5 displays an overview of the risk of bias across all included studies. Of note was that in the RCT studies, bias due to missing outcome data was least problematic, indicating a low dropout rate (six out of seven studies). In contrast, bias due to deviations from intended interventions was most problematic, indicating that, in some studies, participants and trial personnel were not blinded and/or there was no information provided as to whether treatments adhered to intervention protocols (six out of seven studies). In the non-RCT studies, bias in the selection of participants in the study was least problematic, indicating that researchers did not select participants based on participant characteristics after the start of intervention (10 out of 10 studies). In contrast, bias in the measurement of outcomes was most problematic, indicating that participants and outcome assessors were not blinded and/or studies used self-reported measures without clinical interviews (10 out of 10 studies).

Funnel plots and Egger’s test showed no evidence of publication in network meta-analyses. Funnel plots were reasonably symmetric and the result from Egger’s test for sample bias were not significant (*p* = 0.22; see Supplementary Material 4).

## Discussion

In this pairwise and network meta-analyses, the authors assessed data from 17 trials and analyzed the overall and relative effectiveness of eight types of psychological treatments for reducing excessive gaming. The pairwise meta-analysis results indicated large overall effectiveness of psychological treatments in reducing excessive gaming. Although the effectiveness was smaller when compared to the active controls than when compared to the inactive controls, both effect sizes were still large. However, this result needs to be interpreted with caution because there are only seven existing RCT studies and several existing low-quality studies. Network meta-analysis results indicated that a combined treatment of CBT and Mindfulness was the most effective, followed by a combined therapy of CBT and Family intervention, Mindfulness, and then CBT as a standalone treatment, however, this finding was based on a limited number of studies. Overall, the findings suggest that psychological treatments for excessive gaming is promising, but replications are warranted, with additional attention being placed on addressing methodological concerns.

The large effect of psychological treatments in reducing excessive gaming seems encouraging but the stability and robustness of the results need to be confirmed. These authors’ moderator analysis indicated that the effect size of non-RCT studies was not significantly different from that of RCT studies. The authors conducted a moderator analysis using the research quality score (high vs low) and found that research quality did not moderate the treatment effect. The authors also examined publication bias using both funnel plots and Egger’s test and found no evidence of publication bias in network meta-analysis. Because most of the studies included in the review were from Asian countries, the authors examined the generalizability of the finding by testing moderator analysis by regions and found no significant difference of treatment effect sizes between Eastern and Western countries. Finally, although limited studies exist, treatment benefits did not greatly diminish after 1–6 months of follow-ups, indicating possible lasting effects.

Network meta-analysis findings provide some preliminary support for the notion that a combined treatment of CBT and Mindfulness and a combined treatment of CBT and Family intervention are most effective in addressing individuals’ gaming behaviors. These combined therapies were significantly more effective than the CBT standalone approach. CBT has been studied and found to be highly effective in addiction treatment—particularly in reducing excessive gaming due to its attention to stimulus control and cognitive restructuring^13^. However, adding Mindfulness and family intervention may have been more effective than CBT alone, given that gaming is affected not only by individual characteristics, but also external stress or family factors.

Mindfulness generally focuses on helping individuals to cope with negative affective states through mindful reappraisal and aims to reduce stress through mindful relaxation training. The effectiveness of Mindfulness has been validated in other substance and behavioral addiction studies such as alcohol^37^, gambling^38^, and Internet^39^ addiction treatments. Indulging in excessive gaming is often associated with the motivation to escape from a stressful reality^40^, and mindful exercises are likely to help gamers not depend on gaming as a coping strategy.

Because excessive gaming is often entangled with family environments or parenting-related concerns—particularly with adolescents, addressing appropriate parent–adolescent communication and parenting styles within excessive gaming interventions are likely to increase treatment efficacy^41–43^. Based on a qualitative study focused on interviews with excessive gamers^43^, and per reports from interviewed gamers, parental guidance to support regulatory control and encouragement to participate in other activities are important factors to reduce excessive gaming. However, at the same time, if parents excessively restrict their children’s behavior, children will feel increased stress and may further escape into the online world through gaming^44^ as a means of coping with their stress. Our study indicates that appropriate communication among parents and adolescents in addition to parenting styles with respect to game control must be discussed in treatment. However, because only two studies examined the top two ranked combined interventions within this paper, such findings warrant replication.

### Limitations and future directions

These authors identified methodological limitations and future directions in the reviewed studies, which include the following. The authors included non-RCTs to capture data on emerging treatments, but a lack of RCT studies contributes to this paper’s identified methodological concerns. Of 17 studies included, seven were RCT studies and 10 were non-RCT studies. The lack of RCT studies has been repeatedly mentioned in previous review studies^17,18^. In fact, one of the two identified reviews^17^ made the criticism that even CBT (the most widely studied treatment for excessive gaming) was mostly conducted in non-RCT studies, which was commensurate with this paper’s data (only one out of four CBT studies included in this review is a RCT). Including non-RCTs may be likely to increase selection bias by employing easily accessible samples and assigning participants with more willingness (which is an indicator of better treatment outcome) to intervention groups. Selection bias may have increased the effect size of treatments than what is represented in reality and may limit the generalizability of this finding. Thus, more rigorous evaluation through RCTs is necessary in future studies.

While there are concerns surrounding assessment tools, given that all included studies used self-report measures without clinical interviews, this may lead to inaccurate results due to perceived stigma. Additionally, 11 self-reported measurement tools were employed in the included studies—and some of those tools may have poor sensitivity or specificity. A previous narrative review^45^ and a recent meta-analytic review^46^ suggested that the Game Addiction Scale-7, Assessment of Internet and Computer Addiction Scale-Gaming, Lemmens Internet Gaming Disorder Scale-9, Internet Gaming Disorder Scale 9- Short Form, and Internet Gaming Disorder Test-10 have good internal consistency and test–retest reliability. Thus, there is a need for studies to employ clinical interviews and self-report measures with good psychometric features.

Many studies in this included review did not describe whether participants and experimenters were blinded and there was no information about whether treatments adhered to intervention protocols. Although blinding of participants and personnel may be impossible in most psychotherapy studies, it is crucial to evaluate possible performance biases such as social desirability. Also, a fidelity check by content experts is needed to confirm whether treatments adhered to intervention protocols.

Finally, future studies need to examine treatment efficacy in treating both excessive gaming and its comorbid psychiatric symptoms. Internet/gaming addiction has been reported to have a high comorbidity with attention deficit hyperactivity disorder, depression, anxiety, and other substance abuse^47,48^. Our results showed that CBT, BT, and BT + Mindfulness may be effective in reducing depression or anxiety symptoms of excessive gamers. However, other psychological and/or pharmacological treatments such as CBT + Bupropion or Bupropion as a standalone treatment have been also reported as potentially effective treatments for excessive gamers with major depressive disorder^49,50^. Thus, it would be worthwile to examine efficacy of treatments on excessive gamers with dual diagnoses.

## Conclusion

TO the best of the authors’ knowledge, this is the first pairwise meta-analytic and network meta-analytic study that examined the overall effectiveness of psychological treatments and compared the relative effectiveness of diverse treatment options for excessive gaming. Although the authors intentionally used network meta-analysis because of its usefulness in comparing relative effectiveness of currently existing literature, this finding should be interpreted with caution due to the small number of studies. However, as previously indicated, the global prevalence of excessive gaming highlights the need for greater attention to this topic. Studies focused on the effectiveness of diverse gaming interventions help meet the call for further inquiry and study on this topic placed by the DSM-5^7^, and allow greater advances to be made in treating individuals who may have difficulty controlling excessive gaming habits. As such, this study can provide preliminary support for beneficial treatment interventions for excessive gaming as well as recommendations for more rigorous studies to be directed at helping those who have excessive gaming habits.

## Supplementary Information

Supplementary Information.

## Data Availability

The datasets used and/or analyzed during the current study are available from the corresponding author upon reasonable request.
