# Supplementary material for: Psychological treatments for excessive gaming: a systematic review and meta-analysis
Source: Sci Rep. 2022 Nov 28;12:20485. doi: 10.1038/s41598-022-24523-9 (PMC9705304; doi:10.1038/s41598-022-24523-9)
Supplement: Supplementary file 1 — Supplementary Information. [file 41598_2022_24523_MOESM1_ESM.docx]

**Supplement 1.** PRISMA NMA checklist of items to include when reporting a systematic review involving a network meta-analysis

| **Section/Topic** | **Item #** | **Checklist Item** | **Location where item is reported** |
| --- | --- | --- | --- |
| **TITLE** |  |  |  |
| Title | 1 | Identify the report as a systematic review *incorporating a network meta-analysis (or related form of meta-analysis).* | Title page |
| **ABSTRACT** |  |  |  |
| Structured summary | 2 | Provide a structured summary including, as applicable: Background, Methods, Results, Discussion/Conclusions, Other | Abstract |
| **INTRODUCTION** |  |  |  |
| Rationale | 3 | Describe the rationale for the review in the context of what is already known*, including mention of why a network meta-analysis has been conducted.* | Introduction |
| Objectives | 4 | Provide an explicit statement of questions being addressed, with reference to participants, interventions, comparisons, outcomes, and study design (PICOS). | Introduction |
| **METHODS** |  |  |  |
| Protocol and registration | 5 | Indicate whether a review protocol exists and if and where it can be accessed (e.g., Web address); and, if available, provide registration information, including registration number. | Method |
| Eligibility criteria | 6 | Specify study characteristics (e.g., PICOS, length of follow-up) and report characteristics (e.g., years considered, language, publication status) used as criteria for eligibility, giving rationale. *Clearly describe eligible treatments included in the treatment network and note whether any have been clustered or merged into the same node* | Identification and Selection of Studies |
| Information sources | 7 | Describe all information sources (e.g., databases with dates of coverage, contact with study authors to identify additional studies) in the search and date last searched. | Identification and Selection of Studies |
| Search | 8 | Present full electronic search strategy for at least one database, including any limits used, such that it could be repeated. | Identification and Selection of Studies & Supplementary material 2 |
| Study selection | 9 | State the process for selecting studies (i.e., screening, eligibility, included in systematic review, and, if applicable, included in the meta-analysis). | Figure 1 |
| Data collection process | 10 | Describe method of data extraction from reports (e.g., piloted forms, independently, in duplicate) and any processes for obtaining and confirming data from investigators. | Supplementary material 3 |
| Data items | 11 | List and define all variables for which data were sought (e.g., PICOS, funding sources) and any assumptions and simplifications made. | Risk of Bias and Data Extraction |
| **Geometry of the network** | **S1** | Describe methods used to explore the geometry of the treatment network under study and potential biases related to it. This should include how the evidence base has been graphically summarized for presentation, and what characteristics were compiled and used to describe the evidence base to readers. | Data Analysis |
| Risk of bias within individual studies | 12 | Describe methods used for assessing risk of bias of individual studies (including specification of whether this was done at the study or outcome level), and how this information is to be used in any data synthesis. | Risk of Bias and Data Extraction |
| Summary measures | 13 | State the principal summary measures (e.g., risk ratio, difference in means). *Also describe the use of additional summary measures assessed, such as treatment rankings and surface under the cumulative ranking curve (SUCRA) values, as well as modified approaches used to present summary findings from meta-analyses.* | Data Analysis |
| Planned methods of analysis | 14 | Describe the methods of handling data and combining results of studies for each network meta-analysis. | Data Analysis |
| **Assessment of Inconsistency** | **S2** | Describe the statistical methods used to evaluate the agreement of direct and indirect evidence in the treatment network(s) studied. Describe efforts taken to address its presence when found. | Data Analysis |
| Risk of bias across studies | 15 | Specify any assessment of risk of bias that may affect the cumulative evidence (e.g., publication bias, selective reporting within studies). | Data Analysis |
| Additional analyses | 16 | Describe methods of additional analyses if done, indicating which were pre-specified. This may include, but not be limited to, the following: Sensitivity or subgroup analyses | Data Analysis |
| **RESULTS** |  |  |  |
| Study selection | 17 | Give numbers of studies screened, assessed for eligibility, and included in the review, with reasons for exclusions at each stage, ideally with a flow diagram. | Included Studies and Their Characteristics |
| **Presentation of network structure** | **S3** | Provide a network graph of the included studies to enable visualization of the geometry of the treatment network. | Figure 3 |
| **Summary of network geometry** | **S4** | Provide a brief overview of characteristics of the treatment network. This may include commentary on the abundance of trials and randomized patients for the different interventions and pairwise comparisons in the network, gaps of evidence in the treatment network, and potential biases reflected by the network structure. | Network Meta-Analysis |
| Study characteristics | 18 | For each study, present characteristics for which data were extracted (e.g., study size, PICOS, follow-up period) and provide the citations. | Included Studies and Their Characteristics & Table 1 |
| Risk of bias within studies | 19 | Present data on risk of bias of each study and, if available, any outcome level assessment. | Risk of Bias & Figure 5 |
| Results of individual studies | 20 | For all outcomes considered (benefits or harms), present, for each study: 1) simple summary data for each intervention group, and 2) effect estimates and confidence intervals. *Modified approaches may be needed to deal with information from larger networks.* | Pairwise Meta-Analysis & Network Meta-Analysis & Figure 4 |
| Synthesis of results | 21 | Present results of each meta-analysis done, including confidence/credible intervals. *In larger networks, authors may focus on comparisons versus a particular comparator (e.g., placebo or standard care), with full findings presented in an appendix. League tables and forest plots may be considered to summarize pairwise comparisons.* If additional summary measures were explored (such as treatment rankings), these should also be presented. | Figure 2 & Table 3 |
| **Exploration for inconsistency** | **S5** | Describe results from investigations of inconsistency. This may include such information as measures of model fit to compare consistency and inconsistency models, *P* values from statistical tests, or summary of inconsistency estimates from different parts of the treatment network. | Included Studies and Their Characteristics |
| Risk of bias across studies | 22 | Present results of any assessment of risk of bias across studies for the evidence base being studied. | Supplementary material 4 |
| Results of additional analyses | 23 | Give results of additional analyses, if done (e.g., sensitivity or subgroup analyses, meta-regression analyses*, alternative network geometries studied, alternative choice of prior distributions for Bayesian analyses,* and so forth). | Moderator Analysis & Table 2 |
| **DISCUSSION** |  |  |  |
| Summary of evidence | 24 | Summarize the main findings, including the strength of evidence for each main outcome; consider their relevance to key groups (e.g., healthcare providers, users, and policymakers). | Discussion |
| Limitations | 25 | Discuss limitations at study and outcome level (e.g., risk of bias), and at review level (e.g., incomplete retrieval of identified research, reporting bias). *Comment on the validity of the assumptions, such as transitivity and consistency. Comment on any concerns regarding network geometry (e.g., avoidance of certain comparisons).* | Discussion |
| Conclusions | 26 | Provide a general interpretation of the results in the context of other evidence, and implications for future research. | Conclusion |
| **FUNDING** |  |  |  |
| Funding | 27 | Describe sources of funding for the systematic review and other support | Funding sources |

*Note.* PRISMA NMA = Preferred Reporting Items for Systematic Reviews and Meta-Analyses Network Meta-Analysis

**Supplement 2.** Search strategy

**Web of Science 10/31/22**

|  | (game OR games OR gaming OR internet game OR internet games OR internet gaming OR video game OR video games OR video gaming OR computer game OR computer games OR computer gaming OR online game OR online games OR online gaming) AND (addiction OR addictions OR disorder OR disorders OR problem OR problems OR problematic OR disease OR diseases OR excessive OR pathological OR addicted) AND (treatment OR treatments OR intervention OR interventions OR efficacy OR effectiveness OR effective OR clinical OR therapy OR therapies) | 196 |
| --- | --- | --- |

**ProQuest 10/31/22**

|  | ("game" OR "games" OR "gaming" OR "internet game" OR "internet games" OR "internet gaming" OR "video game" OR "video games" OR "video gaming" OR "computer game" OR "computer games" OR "computer gaming" OR "online game" OR "online games" OR "online gaming") AND ("addiction" OR "addictions" OR "disorder" OR "disorders" OR "problem" OR "problems" OR "problematic" OR "disease" OR "diseases" OR "excessive" OR "pathological" OR "addicted") AND ("treatment" OR "treatments" OR "intervention" OR "interventions" OR "efficacy" OR "effectiveness" OR "effective" OR "clinical" OR "therapy" OR "therapies") | 291 |
| --- | --- | --- |

**PubMed 10/31/22**

|  | (("game"[title] OR "games"[title] OR "gaming"[title] OR "internet game"[title] OR "internet games"[title] OR "internet gaming"[title] OR "video game"[title] OR "video games"[title] OR "video gaming"[title] OR "computer game"[title] OR "computer games"[title] OR "computer gaming"[title] OR "online game"[title] OR "online games"[title] OR "online gaming"[title]) AND ("addiction"[title] OR "addictions"[title] OR "disorder"[title] OR "disorders"[title] OR "problem"[title] OR "problems"[title] OR "problematic"[title] OR "disease"[title] OR "diseases"[title] OR "excessive"[title] OR "pathological"[title] OR "addicted"[title])) AND ("treatment"[title] OR "treatments"[title] OR "intervention"[title] OR "interventions"[title] OR "efficacy"[title] OR "effectiveness"[title] OR "effective"[title] OR "clinical"[title] OR "therapy"[title] OR "therapies"[title]) | 205 |
| --- | --- | --- |

**Scopus 10/31/22**

|  | ("game" OR "games" OR "gaming" OR "internet game" OR "internet games" OR "internet gaming" OR "video game" OR "video games" OR "video gaming" OR "computer game" OR "computer games" OR "computer gaming" OR "online game" OR "online games" OR "online gaming") AND ("addiction" OR "addictions" OR "disorder" OR "disorders" OR "problem" OR "problems" OR "problematic" OR "disease" OR "diseases" OR "excessive" OR "pathological" OR "addicted") AND ("treatment" OR "treatments" OR "intervention" OR "interventions" OR "efficacy" OR "effectiveness" OR "effective" OR "clinical" OR "therapy" OR "therapies") AND ( LIMIT-TO ( DOCTYPE,"ar" ) ) | 241 |
| --- | --- | --- |

**PsycINFO 10/31/22**

|  | (Title: game OR Title: games OR Title: gaming OR Title: internet game OR Title: internet games OR Title: internet gaming OR Title: video game OR Title: video games OR Title: video gaming OR Title: computer game OR Title: computer games OR Title: computer gaming OR Title: online game OR Title: online games OR Title: online gaming) AND (Title: addiction OR Title: addictions OR Title: disorder OR Title: disorders OR Title: problem OR Title: problems OR Title: problematic OR Title: disease OR Title: diseases OR Title: excessive OR Title: pathological OR Title: addicted) AND (Title: treatment OR Title: treatments OR Title: intervention OR Title: interventions OR Title: efficacy OR Title: effectiveness OR Title: effective OR Title: clinical OR Title: therapy OR Title: therapies) | 133 |
| --- | --- | --- |

**Dbpia 10/31/22**

|  | Title: Game\Internet Game\Video Game\Computer Game <AND> Title: Addiction\Disorder\Problem\Disease\immersion\Pathology\Overdependence <AND> Title: Treatment\Effect\Effectiveness\Clinical\Intervention\Counseling\ program | 144 |
| --- | --- | --- |

**RISS 10/31/22**

|  | Title: Game\Internet Game\Video Game\Computer Game <AND> Title: Addiction\Disorder\Problem\Disease\immersion\Pathology\Overdependence <AND> Title: Treatment\Effect\Effectiveness\Clinical\Intervention\Counseling\ program | 261 |
| --- | --- | --- |

**Supplement 3.** Example of intervention type classification

| Study reference:  Liu, L., Potenza, M. N., Lacadie, C. M., Zhang, J. T., Yip, S. W., Xia, C. C., ... & Fang, X. Y. (2020). Altered intrinsic connectivity distribution in internet gaming disorder and its associations with psychotherapy treatment outcomes. *Addiction Biology*, *26*(2), e12917. | | | |
| --- | --- | --- | --- |
| **Authors’ final classification decision** | | Combined therapy of Behavioral Therapy and Mindfulness based on Zhang and colleagues (2016) | |
| **First node by SL and DJ** | | BT + Mindfulness | |
| **Second node by JK** | | BT + Mindfulness | |
| **Treatment Category** | BT + mindfulness | **Name of treatment** | Craving Behavioral Intervention (CBI) |
| **# of sessions** | 6 | **Hours per session** | 2.5 to 3 hours |
| **Individual or Group** | Group (8-9 participants) | **Follow-up period** | 3 months & 6 months |
| **Program description** 2.3 \| Craving behavioral intervention *(ps. 1-3)*  The Craving Behavioral Intervention (CBI) was developed on theoretical foundations for behavioral interventions in addictions (Brand et al., 2016; Dong & Potenza, 2014) the craving framework of boundary conditions, and the fulfillment of psychological needs for Internet use (Suler, 1999), as described in previous publications (Zhang et al., 2016a; Zhang et al., 2016b). Because craving may contribute importantly to the development and maintenance of Internet Gaming Disorder (IGD), interventions that help participants cope with and reduce craving may promote positive therapeutic outcomes and prevent relapse (Brand et al., 2016). The group intervention was conducted once per week and included six 2.5 hour sessions, with eight to nine IGD participants in each group. The details of the CBI are included in the Supporting Information.  The CBI for IGD was developed on the theoretical basis of behavioral interventions for IGD (Brand et al., 2016; Dong & Potenza, 2014), the craving framework of boundary conditions, and the fulfillment of psychological needs for Internet use (Suler, 1999). The CBI was developed to help individuals with IGD: 1) recognize subjective craving and its relationship with Internet-gaming behaviors; 2) reduce craving through ameliorating the salience of gaming cues and **craving-related irrational beliefs,** withdrawal symptoms and other negative affects; 3) enhance self-monitoring and control for craving through time management training; and, 4) relieve fulﬁllment of psychological needs through Internet use and attenuate the relationship between craving and gaming behaviors through training of healthy coping skills.  *(Additional document: Zhang 2016a, p. 2)*  The topics for each session focused on: 1) understanding and perceiving subjective craving for Internet gaming, listing gaming-related scenes that might trigger craving, and Mindfulness training for addressing gaming-cue-induced craving and tension; 2) **recognizing and testing irrational beliefs** regarding craving and exploring other possible inferences; 3) detecting emotions following craving and learning and utilizing effective approaches for regulating craving, including Mindfulness training to experience and relieve craving-related emotions; 4) shifting participants' fulﬁllment of psychological needs from the Internet to reality and building adaptive relationships with peers; 5) learning time management and skills training for coping with craving; 6) maintaining the effectiveness of the intervention through reviewing and practicing, and setting up adaptive and positive plans for daily life in the future. In addition, **Mindfulness training was self-administered every time participants experienced craving outside the intervention hours as an assignment.** | | | |
| Additional reference: Zhang, J. T., Yao, Y. W., Potenza, M. N., Xia, C. C., Lan, J., Liu, L., ... & Fang, X. Y. (2016). Altered resting-state neural activity and changes following a craving behavioral intervention for Internet gaming disorder. *Scientific Reports*, *6*(1), 1-8. | | | |

**Supplement 4.**

Comparison-adjusted funnel plot for the network meta-analysis


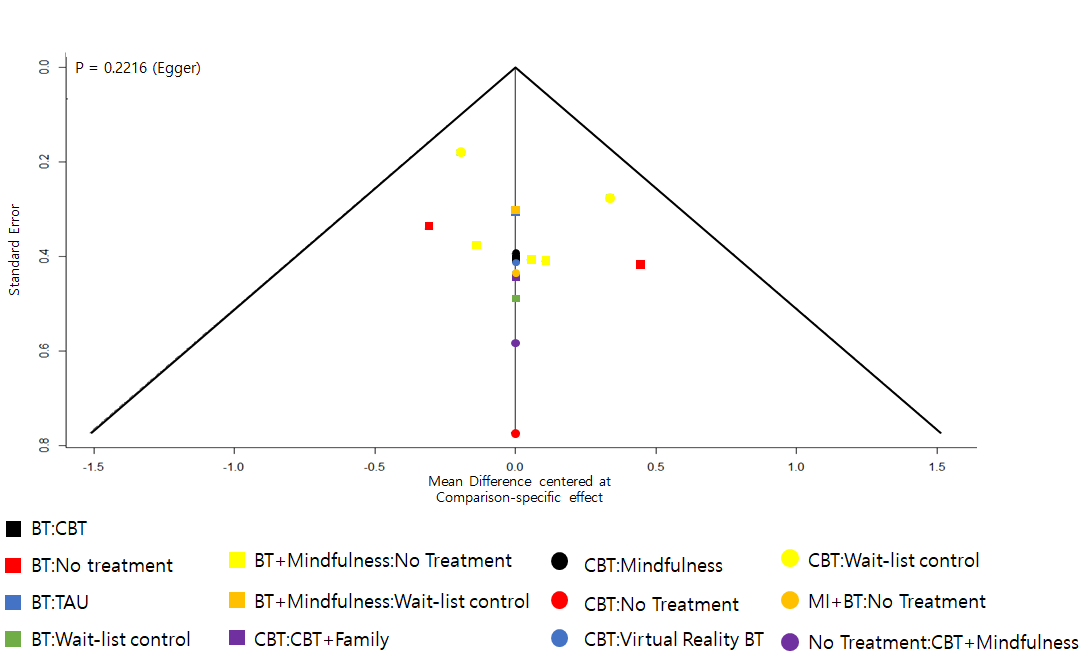


*Note*. BT = Behavioral Therapy; CBT = Cognitive Behavioral Therapy; Family = Family Intervention; MI = Motivational Interviewing; TAU = Treatment As Usual
